# Patterns and cost of care according to keratinocyte cancer risk stratification in a volunteer population screening clinic: Real‐world data from the TRoPICS study

**DOI:** 10.1111/ajd.14054

**Published:** 2023-04-24

**Authors:** Ruby Chia‐Lin Lee, Upekha Liyanage, Kirsty Fry, Susan Brown, Lena von Schuckmann, Lynda Spelman, H. Peter Soyer, Rachel E. Neale, Louisa G. Gordon, David C. Whiteman, Catherine M. Olsen, Monika Janda, Kiarash Khosrotehrani

**Affiliations:** ^1^ Frazer Institute, The University of Queensland Brisbane Queensland Australia; ^2^ QIMR Berghofer Medical Research Institute Brisbane Queensland Australia; ^3^ Queensland Institute of Dermatology Queensland Skin and Cancer Foundation Brisbane Queensland Australia; ^4^ Dermatology Research Centre Frazer Institute, The University of Queensland Brisbane Queensland Australia; ^5^ Centre of Health Services Research The University of Queensland Brisbane Queensland Australia

**Keywords:** chemoprophylaxis, keratinocyte carcinoma, risk stratification

## Abstract

**Background:**

Risk prediction tools have been developed for keratinocyte cancers (KCs) to effectively categorize individuals with different levels of skin cancer burden. Few have been clinically validated nor routinely used in clinical settings.

**Objectives:**

To assess whether risk prediction tool categories associate with interventions including chemoprophylaxis for skin cancer, and health‐care costs in a dermatologist‐run screening clinic.

**Methods:**

Adult participants who presented to a walk‐in screening facility were invited to participate. A self‐completed KC risk prediction tool was used to classify participants into one of the five risk categories. Participants subsequently underwent full skin examination by a dermatologist. Dermatological interventions and skin cancer‐related medical prescriptions were documented. Total health‐care costs, both to the health‐care system and patients were evaluated.

**Results:**

Of the 507 participants recruited, 5‐fluorouracil cream and nicotinamide were more frequently prescribed in the higher risk groups as chemoprophylaxis (*p* < 0.005). A significant association with high predicted risk was also observed in the use of cryotherapy and curettage and cautery (*p* < 0.05). The average health‐care costs associated with a skin check visit increased from $90 ± 37 (standard deviation) in the lowest risk group to $149 ± 97 in the highest risk group (*p* < 0.0001).

**Conclusions:**

We observed a positive association between higher predicted risk of skin cancer and the prescription of chemoprophylaxis and health‐care costs involved with opportunistic community skin cancer screening. A clinical use of risk stratification may be to provide an opportunity for clinicians to discuss skin cancer prevention and chemoprophylaxis with individual patients.

What This Research Add

- Few skin cancer risk prediction tools have been clinically validated nor routinely used in clinical settings.
- In our study, a positive association was found between higher predicted risk of skin cancer and the prescription of chemoprophylaxis and associated healthcare costs in opportunistic community skin cancer screening.
- A clinical utility of risk stratification may be to provide an opportunity for clinicians to discuss skin cancer prevention and chemoprophylaxis.

## INTRODUCTION

Keratinocyte carcinomas (KCs) are the most common cancers in fair‐skinned populations. They contribute to significant morbidity for patients and cause a large burden on our health‐care system.^1^, ^2^, ^3^ The estimated health expenditure in Australia for KCs was $1.3 billion in 2018–2019, with the highest cancer expenditure attributable to risk factors compared to other types of cancers.^4^

Risk prediction models for KCs have been developed as a way of estimating risks and personalizing preventive interventions. These tools commonly include algorithms based on epidemiological,^5^ demographic,^5^, ^6^, ^7^, ^8^ phenotypic^5^, ^6^, ^7^, ^8^, ^9^ and genetic characteristics^10^, ^11^, ^12^, ^13^, ^14^ of participants. However, few have been validated, routinely used in clinical settings or assessed for clinical implications.

External validation of risk prediction models is important in assessing its clinical utility. In a recent study (Tool for Risk Prediction in Cancers of the Skin [TRoPICS]),^15^ we validated the clinical utility of administering a risk stratification questionnaire^5^ (accessible on https://publications.qimrberghofer.edu.au/p/qimr/qskinriskcalculator) immediately prior to a skin examination and identifying individuals with variable levels of prevalent skin cancer in a community screening setting. We found that the proportion of participants with prevalent KCs diagnosed at a volunteer skin examination increased significantly with the higher risk categories. Thus, the KC risk prediction model was shown to identify individuals with a significant skin cancer burden with good reliability.

Chemoprophylactic therapies have been shown to minimize the risk of developing multiple skin cancers. These therapies include systemic chemoprophylaxis in the forms of oral retinoids and vitamin A analogues, nicotinamide and topical therapies targeting field cancerization of photodamaged skin such as fluorouracil.^16^, ^17^ Currently, clinicians have to rely on their own experience to estimate an individual's future risk of skin cancers in order to identify those at high‐risk who would benefit most from earlier intervention.

To date, no studies have specifically examined the association between predicted risk of skin cancer and the costs of skin cancer prevention and treatment. In this study, we aimed to assess whether chemoprophylaxis prescription, skin cancer interventions and their costs varied according to predicted risk categories in a volunteer dermatologist‐run screening clinic. These findings using real‐world data contribute to the clinical utility of risk stratification tools and may support its use in clinical settings.

## MATERIALS AND METHODS

### Study population and data collection

Data used for this study were collected over 1 week in August 2019 on Hamilton Island, Queensland, a tropical resort island (20° S) along the eastern coast of Australia. The protocol was approved by the University of Queensland Human Research Ethics Committee (approval number: 2019001803).

Details of recruitment have been described previously.^15^ Briefly, a free of charge, temporary clinic was established during the annual Hamilton Island yachting race week. Dermatologists volunteered to conduct skin examinations for any person presenting to the clinic, including competitors and the entire island community. The clinic was advertised across the island and included in advertising for race‐related events prior to and during the week. Each person underwent a full body skin examination, followed by dermatological procedures such as cryotherapy, curette and cautery, diagnostic biopsies of suspicious lesions if applicable. Prescriptions for chemoprophylactic therapies were provided at the time of skin assessment if considered to be of benefit by the dermatologist. These included field‐based topical prescriptions, namely 5‐fluorouracil (5‐FU) cream and 0.05% tretinoin cream, or systemic nicotinamide. The study focused on prescription, interventions and costs incurred during the screening consultation. Follow‐up treatments such as full excision or other interventions as a result of biopsies during the screening clinic were not taken into account.

All people presenting for a skin examination were invited to participate in the study. All participants provided informed consent and completed a paper‐based questionnaire including demographic and medical history, and items of the Keratinocyte Cancer Stratification Tool^5^ before undergoing skin examination by dermatologists who were blinded to the questionnaire responses. Dermatological procedures performed and prescriptions provided by dermatologists were recorded and the participants' risk categories were calculated.

### Statistical analysis

The risk prediction tool^5^ was used to place participants into one of five risk categories, ranging from ‘very much below average’ to ‘very much above average’. The cut‐points were derived from the quintiles of the risk distribution observed in the QSkin Study sample (*n* = 43,794) and reflected the skin cancer risk distribution in Queensland, Australia.^5^, ^18^ We calculated the percentage of participants in each risk category who had undergone each type of intervention or prescription.

Direct health‐care costs associated with an individual's skin examination visit and procedures performed were assessed according to the costs paid by the government per Australian Medical Benefits Schedule (MBS) item numbers (accessible on http://www.mbsonline.gov.au). These were the billing items for specialist attendance, diagnostic biopsy, cryotherapy and serial curettage excision. (See Table S1 for individual MBS item numbers included for calculation). Participants were not charged additional out‐of‐pocket fees.

Medication costs were calculated according to the dispensing fees on the Pharmaceutical Benefits Scheme (PBS) (accessible on http://www.pbs.gov.au) or the recommended retail price for non‐PBS medications, both paid out‐of‐pocket by participants. Annual averages were estimated according to standard dosing regimens of 5‐FU creams, tretinoin creams and nicotinamide. The estimation of medication costs included the costs of new medications prescribed during the skin examination visit, and did not include the costs of ongoing nor pre‐existing prescriptions. (See Table S1 for medication dispensing prices for estimation).

Total health‐care costs for each participant were determined as the sum of all costs above; that is, the costs of the consultation, plus the itemized treatment costs incurred during one visit to the volunteer skin screening clinic, plus the annual costs of skin cancer treatment for each patient. The mean cost within each KC risk score category was calculated.

The Kruskal–Wallis test was used to evaluate whether the interventions, treatments and prescription costs differed significantly between KC risk categories.

## RESULTS

### Study population

Of the 789 people who presented for a skin examination during the screening week, 558 (71%) consented to participate in the study and 507 returned a fully completed questionnaire. Detailed characteristics of the participants have been described previously.^15^ Participants were mostly older than 45 years (59%), with a slight predominance of men (56% male). The majority of participants (91%) were of European ancestry. Almost two thirds of participants (63%) were at ‘below’ or ‘very much below average’ risk, and 37% were at ‘average’, ‘above average’ and ‘very much above average’ risk. Closer to one fourth of participants (28%) has not had a skin checked in the last 3 years, and one third (32%) has had one deliberate skin examination in the last 3 years.

### Treatment and prescription of chemoprophylaxis according to risk categories

Among the 507 participants, 39 (7.7%) were prescribed 5‐FU cream and 33 (6.5%) were prescribed 0.05% tretinoin cream as field‐based therapies; a further 17 (3.3%) participants were prescribed nicotinamide as chemoprophylaxis (Table 1). Cryotherapy was performed for 54 (10.7%) participants, and curettage and cautery for 4 (0.01%) participants. A total of 133 diagnostic biopsies were performed in 94 (18.5%) participants. Most participants (70%) did not receive any interventions nor prescriptions.

**TABLE 1:** Number and percentage (%) of medical prescriptions and dermatological interventions performed in each predicted risk group.

	Very much below average (*n* = 115)	Below average (*n* = 202)	Average (*n* = 41)	Above average (*n* = 108)	Very much above average (*n* = 41)	*p* value
	*n* (%)	*n* (%)	*n* (%)	*n* (%)	*n* (%)	
Nicotinamide	0 (0)	3 (1.5)	3 (7.3)	8 (7.4)	3 (7.3)	0.0036
5‐Fluorouracil cream	0 (0)	6 (3.0)	5 (12.2)	19 (17.6)	9 (22.0)	<0.0001
0.05% Tretinoin cream	7 (6.1)	15 (7.4)	4 (9.8)	6 (5.6)	1(2.4)	0.6815
Cryotherapy	4 (3.5)	21 (10.4)	2 (4.9)	16 (14.8)	11 (26.8)	0.0003
Curette and cautery	1 (0.9)	1 (0.5)	0 (0)	0 (0)	2 (4.9)	0.0379
Diagnostic biopsy^a^	15 (13.0)	38 (18.8)	16 (39.0)	39 (36.1)	25 (61.0)	<0.0001

*Note*: n = number.

^a^ Total number of biopsies performed in each predicted risk group (average number of biopsies per participant).

**FIGURE 1:**
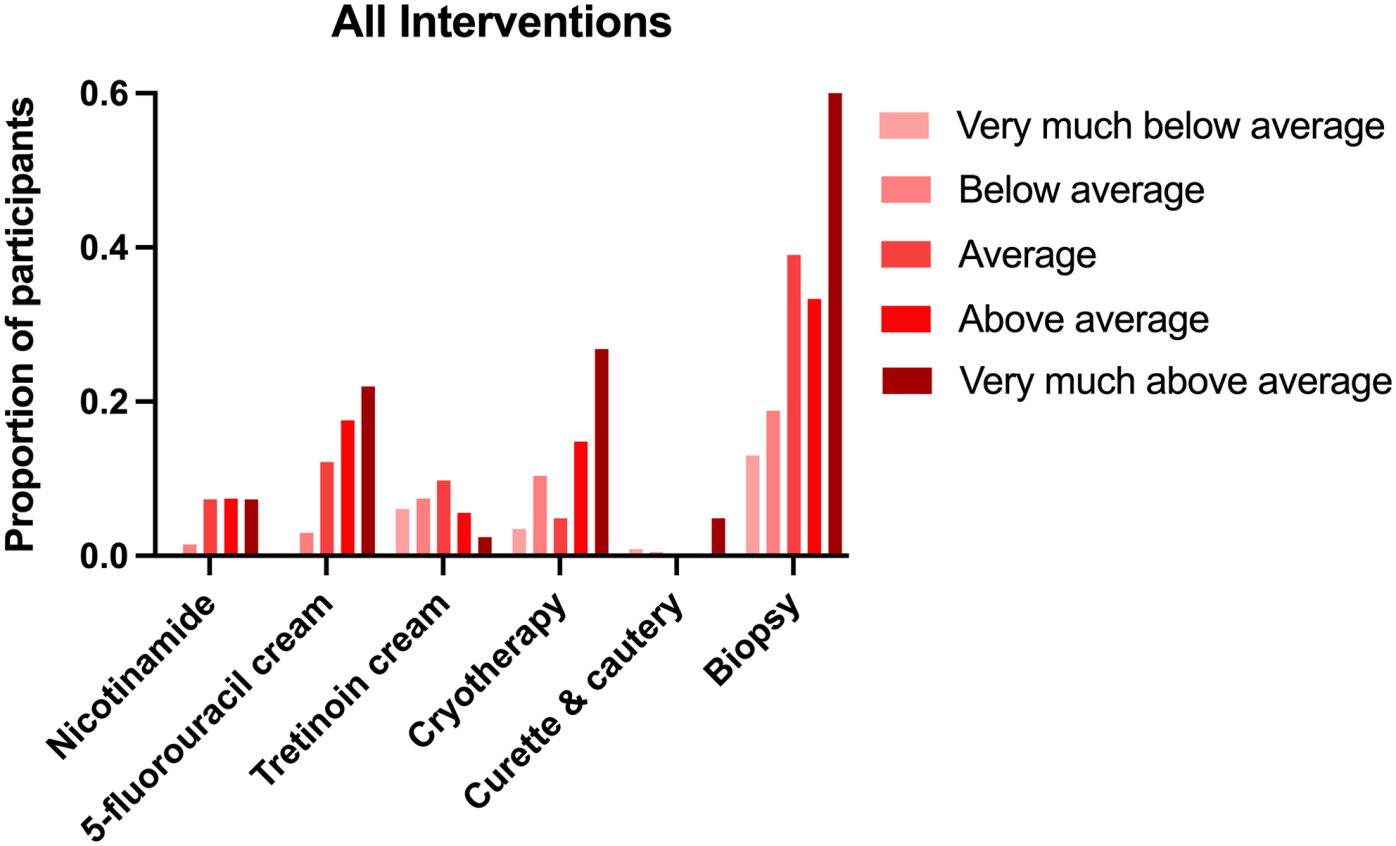
Proportion of participants receiving skin cancer‐related medical interventions and prescriptions in each predicted risk group.

There was a steady increase in the proportion of participants prescribed 5‐FU as predicted risks increased (*p* < 0.05) (Figure 1). 5‐FU was prescribed in <5% of participants who were classified as ‘very much below average’ and ‘below average’ risk, compared to 17.6% in the ‘above average’, and 22.0% in the ‘very much above average’ risk groups (*p* < 0.0001). Nicotinamide prescription was also associated with risk (*p* < 0.05), being prescribed in 1.5% of participants in the ‘below average’ risk group, compared to 7.4% and 7.3% in the ‘above average’, and ‘very much above average’ risk groups respectively (*p* = 0.0036).

Conversely, the prescription of low‐dose (0.05%) tretinoin cream was variable and did not differ significantly across the risk categories (*p* = 0.68).

In contrast, a significant association was observed between skin cancer risk and the frequency of cryotherapy and curettage and cautery (*p* < 0.05). Cryotherapy was performed in 3.5% of participants with ‘very much below average’ risk and 10.4% with ‘below average’ risk, compared to 14.8% in the ‘above average’ group and 26.8% in the ‘very much above average’ risk group (*p* = 0.0003).

### Health‐care costs according to risk categories

The estimated total health‐care costs to patient and government (Medicare and PBS) of skin examination, procedures and medication costs resulting from screening the 507 participants over 1 week was AU$57,415. Overall, the costs associated with a skin check visit (and the associated prescriptions) increased with increasing risk categories for both the benefits paid by the government and out‐of‐pocket medication expenses by patients (Table 2). Mean health‐care costs increased from $90 ± 37 (standard deviation) in the ‘very much below average’ risk group to $149 ± 97 in the ‘very much above average’ risk group (*p <* 0.0001).

**TABLE 2:** Health‐care costs, in Australian Dollars ($AUD), within each predicted risk group.

	Australian medicare billing to government						Patient out‐of‐pocket costs for medications					
	VMBA	BA	A	AA	VMAA	All	VMBA	BA	A	AA	VMAA	All
Total	9452	17,004	3660	9770	4099	43,985	910	3446	1883	5167	2023	13,430
Mean	82	84	89	90	100	87	8	17	46	48	49	26
SD	19	20	27	24	39	24	31	57	91	85	87	68
%	22	39	8	22	9	100	7	26	14	38	15	100

*Note*: % = percentage of total cost.

Abbreviations: A, average; AA, above average; BA, below average; VMAA, very much above average; VMBA, very much below average; SD, standard deviation.

## DISCUSSION

Models to estimate the future risk of developing skin cancers are helpful in identifying those most likely to suffer the greatest morbidity. There is a clinical need to identify individuals at high risk who would benefit the most from earlier interventions.

We classified participants according to their risk of KC using a previously published stratification tool. We found a strong positive association between the predicted risk of KC and prescription of chemoprophylaxis, performance of dermatological procedures and treatment costs associated with undergoing a skin examination. This positive association is consistent with our previous study,^15^ where the number of KCs diagnosed increased with increasing risk. Similarly, an economic evaluation of a prospective international multicentre study of private dermatology clinics showed that the cost of skin cancer screening, from examination and biopsy costs, increased with age and lower skin phototypes, both of which are established skin cancer risk factors.^19^

Few studies have addressed the pattern of chemoprophylaxis prescription for KCs in real‐world settings.^20^, ^21^ Fluorouracil prescription has previously been found to be more common in people aged 50–80 years, in men compared to women, for use on anatomical location away from the face, and prescribed by dermatologists.^21^ Our findings suggest that risk scores developed using participant‐reported data are associated with likelihood of chemoprophylaxis prescription and having procedures performed in a volunteer screening clinic. Prescription patterns identified in our study were expected, where 5‐FU was prescribed for participants in the higher risk groups, who likely had visible field cancerization on examination.^17^ On the other hand, there was no clear nor statistically significant pattern in the prescription of low‐dose tretinoin cream. This could be due to its perceived lower efficacy in treating actinic keratosis in the high‐risk group as opposed to topical fluorouracil.^22^ Due to the nature of the clinic, clinicians did not prescribe other forms of chemoprevention that required regular biochemistry monitoring such as oral retinoids.

It is encouraging that without having the results of the risk scores, dermatologists still targeted the most effective interventions to the high‐risk group. However, many in the highest risk category were not prescribed any chemoprophylaxis. The benefit of the risk score in routine practice may be to facilitate the discussion with patients about the need for intervention. In particular, the use of risk stratification tools is commonplace in primary care settings (e.g. for cardiovascular disease and diabetes) where a large proportion of skin cancers are managed.^23^ We contend that such tools could easily be adopted in primary care, with a view to prompting a consideration of prescribing chemoprophylaxis for high‐risk patients in that setting. Indeed, compliance with chemoprevention is always of concern and clear communication has been shown to improve compliance and outcomes.^24^

The strengths of this study are the prospective enrolment of participants with a broad distribution of KC risk scores. All medications and interventions (of surgical and non‐surgical treatments of premalignant lesions) were advised by specialist dermatologists, who were blinded to the survey responses of the participants. As such, the prescription patterns of the dermatologists were not subject to bias. The limitation of this study was the broad distribution of risk scores, as participants were not representative of the general Queensland population as in the QSkin study.^18^ Instead, many of our recruited participants were from the tropics, and were highly exposed to the sun, of younger age and with a good ability to tan.^5^, ^15^ This was reflected by the risk distribution that was not in keeping with the general population.^5^ Furthermore, 41% of the participants were less than 45 years of age, in comparison to the QSkin study cohort being aged 40–69 years old. However, age is only one of the risk stratification items in the KC risk calculator and many of the participants had other risk factors that put them at an overall high risk. Given the risk prediction tool chosen was based on a population in Queensland, with the highest reported incidence of skin cancers in the world, the data may not be applicable to other Caucasian populations elsewhere.^25^

Many cost‐effectiveness studies have been performed to assess the value of skin cancer management in various health systems, institutions and specialized clinics.^26^, ^27^, ^28^, ^29^ Few have assessed the health‐care costs by skin cancer risk stratification. A UK study compared the cost‐effectiveness of various surveillance strategies for melanoma, according to a self‐assessment stratification tool.^30^ To the best of our knowledge, no other studies have estimated costs associated with community‐based screening of skin cancers based on risk stratification. Our estimates may allow projecting the cost of community‐based screening for future clinical trials or public health initiatives associated with skin cancer screening.

Previous cost‐analysis studies from Australia primarily focused on the costs of medical attendances, surgical procedures and histopathology services.^3^, ^29^, ^31^ The estimated costs in our study focused on chemoprophylaxis, in addition to consultations and skin biopsies typically considered in a screening setting. Health‐care cost analysis did not include additional out‐of‐pocket fees for prevention (such as sunscreen, sunglasses and hats), consultation or procedures that were incurred in follow‐up settings after the screening, nor the cost of histology reading by pathology companies. However, the health‐care costs calculated were still reflective of the general costs billed to the Australian Medicare system, and the minimum out‐of‐pocket expenses of medications for patients. Importantly, these costs could be underestimated as our study only focused on new prescription of chemopreventive medications and did not take into account the ongoing prescription of nicotinamide and chemopreventive topical therapies for some participants. However, chemoprophylaxis was still prescribed to participants who have had previous skin checks. Furthermore, given our participants were recruited from a community‐based volunteer‐screening clinic, where at least half (60%) had less than two deliberate skin examinations in the last 3 years, the estimation of health‐care costs is likely to be accurate and not biased by past prescriptions.

In conclusion, our study demonstrates a positive association between predicted risk of skin cancer and the frequency of prescription of chemoprophylaxis and procedures performed in a volunteer‐based skin cancer screening. The tool also provides an estimation of future health‐care costs associated with opportunistic community skin cancer screening. Higher risk groups were associated with higher health‐care costs. Given the risk prediction tool is easy to use,^15^, ^32^ its implementation may serve as an opportunity for clinicians to discuss skin cancer prevention with patients to increase preventive efforts.

## AUTHOR CONTRIBUTIONS

Conception of the work (Ruby Chia‐Lin Lee and Kiarash Khosrotehrani); design of the work (Kiarash Khosrotehrani, Rachel E. Neale, Monika Janda, David C. Whiteman, Louisa G. Gordon, Catherine M. Olsen, Ruby Chia‐Lin Lee, Lynda Spelman, H. Peter Soyer and Susan Brown); acquisition of the data (Kiarash Khosrotehrani, Lena von Schuckmann, Kirsty Fry and Ruby Chia‐Lin Lee); analysis of work (Ruby Chia‐Lin Lee, Upekha Liyanage, Kiarash Khosrotehrani and Kirsty Fry); drafting the work (Ruby Chia‐Lin Lee, Upekha Liyanage and Kiarash Khosrotehrani), revising the work for important intellectual content (all authors). Final approval of the version to be published (all authors). Kiarash Khosrotehrani and Ruby Lee as well as all authors agree to be accountable for all aspects of the work in ensuring that questions related to the accuracy or integrity of any part of the work are appropriately investigated and resolved.

## FUNDING INFORMATION

Dr Kiarash Khosrotehrani was supported by the Advance Queensland Clinical Research Fellowship. Dr H. Peter Soyer holds an NHMRC MRFF Next Generation Clinical Researchers Program Practitioner Fellowship (APP1137127). Dr David C. Whiteman was supported by a NHMRC Research Fellowship 1155413. The QSkin Study is supported by NHMRC Grants 1073898, 1063061 and 1185416. Dr Ruby Chia‐Lin Lee was supported by the University of Queensland Research Training Program Tuition Fee Offset Scholarship and Research Training Program Stipend Scholarship.

## CONFLICT OF INTEREST STATEMENT

Dr Lee, Dr Liyanage, Ms Brown, Ms Fry, Dr von Schuckmann, Dr Spelman, Dr Neale, Dr Olsen and Dr Janda have no conflict of interest to disclose. Dr Khosrotehrani is a Co‐Editor‐in‐Chief for the *Australasian Journal of Dermatology*. Dr Soyer is a shareholder of MoleMap NZ Limited and e‐derm consult GmbH, and undertakes regular teledermatological reporting for both companies. Dr Soyer is a Medical Consultant for Canfield Scientific Inc, MoleMap Australia Pty Ltd, Blaze Bioscience Inc and a Medical Advisor for First Derm. Dr Whiteman reports grants from National Health and Medical Research Council of Australia; other from Skin Cancer College Australasia, during the conduct of the study; and personal fees from Pierre Fabre, outside the submitted work.

## ETHICS STATEMENT

All study procedures were approved by the University of Queensland Human Research Ethics Committee (2019001803) and performed in accordance with the World Medical Association Declaration of Helsinki. The patients in this article have given written informed consent to publication of their case details.

## Supporting information


Table S1.

## Data Availability

All data generated or analysed during this study are included in this published article (and its supplementary information files).
