# Supplementary material for: Patterns and cost of care according to keratinocyte cancer risk stratification in a volunteer population screening clinic: Real‐world data from the TRoPICS study
Source: Australas J Dermatol. 2023 Apr 24;64(3):389–96. doi: 10.1111/ajd.14054 (PMC10952310; doi:10.1111/ajd.14054)
Supplement: Supplementary file 1 — Table S1. [file AJD-64-389-s001.docx]

Supplementary Table 1. Fees of the Australian Medical Benefits Schedule item numbers. Medication costs according to the Pharmaceutical Benefits Scheme and recommended retail price. ($ in Australian Dollars).

| **Australian Medicare Billing Items** | **85% Benefit paid** |  |
| --- | --- | --- |
| 104 - Specialist attendance | $76.80 |  |
| 30071^‡^ - Biopsy for diagnostic purposes | $46.20 |  |
| 30196^‡^ - Cryotherapy and serial curettage excision | $111.65 |  |
| ^‡^ subject to Multiple Operation Rule |  |  |
| **Medical prescriptions** | **Dispensed price** | **Annual averages** |
| Tretinoin 0.5mg/g (ReTrieve) cream^§^, 50g | $65.99 | $129.98 |
| Fluorouracil 5% (Efudix) cream^†^, 20g | $66.21 | $132.42 |
| Nicotinamide 500mg tablet^‡^, 60 tablets | $19.49 | $233.88 |

^§^ estimated two tubes used per year as daily application to the face

^†^ estimated two tubes used per year as a standard course of field treatment to face and bilateral forearms

^‡^ annual averages according standard chemoprophylactic dosing of 500mg twice a day
